# Some Remarkable Figures and Facts

**Published:** 1916-07-01

**Authors:** 


### THE ROYAL NATIONAL PENSION FUND FOR NURSES.

### Some Remarkable Figures and Facts.

A full report of the proceedings at the twenty-ninth annual general meeting of the Royal National Pension Fund for Nurses, held at the Royal Society of Arts, John Street, Adelphi, on the 22nd ultimo, under the presidency of Sir Everard Hambro, K.C.V.O., the chairman of the Council, will be found in this week's issue of *The Nursing Mirror.* The meeting was full of interest, and brought out some striking facts which must find a place in The Hospital, seeing that this Fund is one of its children. The progress of this Fund has been remarkable, and the invested funds on December 31, 1915, amounted to no less than £2,012,000. The late Mr. Junius S. Morgan, and the great firm of which he was once the head, have always upheld it, and it is fitting that large sums should have been invested in approved American securities. During 1915 the Council made an offer, which was accepted by the Government, to whom the Fund sold £530,000 worth of American securities, and invested the whole of that large sum of money in War Stock. The Fund has at present £758,000, or 37 per cent. of its total investments, in English Government securities. The Government have recognised the great value and importance to the nation of the Pension Fund by refunding the income tax paid by the Fund during the last three years. The amount returned has been placed to the reserve fund, which now exceeds £102,000. The expenses of management are £6 9s. 7d. per cent. on the premiums received, a moderate figure indeed. Two thousand nurses are to-day drawing annuities from the Pension Fund to the amount of £5,000 a month. Is it surprising that Mr. Dewey, out of his fulness of knowledge, which is well-nigh unique, in answering Florence Nightingale's question in her letter of October 1879 (see page 330), "What can our nurses do for themselves?" replied, "Yes, the nursing profession to-day is unquestionably the noblest and best example of thrift in this country." We agree, but Florence Nightingale would have felt sorry indeed to know that when every facility has been given through this Fund to nurses to provide for their future, relatively few of them, as Mr. Dewey added, have taken the trouble to embrace the opportunity placed within their reach. We are proud of our child, the Royal National Pension Fund for Nurses, and in commending it to the attention of every trained nurse we congratulate the Council upon the gratifying success which they were able to report to the nurses at the meeting on the 22nd ultimo.

